# Microscopic Characterization of Bioactivate Implant Surfaces: Increasing Wettability Using Salts and Dry Technology

**DOI:** 10.3390/ma14102608

**Published:** 2021-05-17

**Authors:** Francesco Gianfreda, Donato Antonacci, Carlo Raffone, Maurizio Muzzi, Valeria Pistilli, Patrizio Bollero

**Affiliations:** 1 Department of Industrial Engineering, University of Rome “Tor Vergata”, 00133 Rome, Italy; 2 Independent Researcher, 70121 Bari, Italy; donat.antonacci@gmail.com; 3 Independent Researcher, 00198 Rome, Italy; raffonecarlo@hotmail.it (C.R.); valeria.pistilli93@gmail.com (V.P.); 4 Department of Science, University Roma Tre, Viale G. Marconi, 446, 00146 Rome, Italy; maurizio.muzzi@uniroma3.it; 5 Department of System Medicine, University of Rome “Tor Vergata”, 00133 Rome, Italy; patrizio.bollero@ptvonline.it

**Keywords:** surface energy, hydrophilicity, contact angle, wettability, bioactivate implant surfaces, surface chemistry, ultra-hydrophilic, nanostructures, exsiccation layer

## Abstract

The surface topography of dental implants plays an important role in cell-surface interaction promoting cell adhesion, proliferation and differentiation influencing osseointegration. A hydrophilic implant leads to the absorption of water molecules and subsequently promotes the adhesion of cells to the implant binding protein. Dried salts on the implant surfaces allow one to store the implant surfaces in a dry environment while preserving their hydrophilic characteristics. This process has been identified as “dry technology”. The aim of the present study is to describe from a micrometric and nanometric point of view the characteristics of this new bioactivated surface obtained using salts dried on the surface. Topographic analysis, energy-dispersive X-ray spectroscopy, and contact angle characterization were performed on the samples of a sandblasted and dual acid-etched surface (ABT), a nanosurface (Nano) deriving from the former but with the adding of salts air dried and a nanosurface with salts dissolved with distilled water (Nano H_2_O). The analysis revealed promising results for nanostructured surfaces with increased wettability and a more articulated surface nanotopography than the traditional ABT surface. In conclusion, this study validates a new promising ultra-hydrophilic nano surface obtained by sandblasting, double acid etching and surface salt deposition using dry technology.

## 1. Introduction

In recent years, a large number of implant surfaces have been developed with the aim of improving the osseointegration process and decreasing implant failures [1]. Each new implant surface presents peculiarities in function of roughness, wettability, topography and surface chemistry with the aim of being bioactive towards the bone [2]. A surface can be defined as “bioactive” when is able to create a bond between living cells and the implanted material [3].

Surfaces play a fundamental role in the interaction with cells and therefore they can be modified with the aim of increasing the adhesion of cells from the bloodstream, enhancing cell proliferation and differentiation of mesenchymal cells into osteoblasts (e.g., grit blasting, acid-etching, anodization, calcium phosphate coating, and plasma-spraying) [4,5,6,7]. Some animal studies have shown better bone-to-implant contact during initial healing than machined implants and the results have subsequently been validated by human studies as well [8,9,10,11,12,13].

In a literature review by Albrektsson and Wennerberg [14] it was reported that implant surfaces can be characterized by multiple aspects such as roughness, chemical composition, physical properties such as wettability and crystallinity and mechanical properties.

The surface topography was developed at the micro (1–10 µm) and nano (1–100 nm) levels. From a micrometric point of view, the surface roughness seems to guarantee greater biomechanical performance while the nanostructures determining a nano-roughness guarantee better adhesion of the integrins to the implant in the initial stages of healing [15]. 

Surfaces have been classified by Wennerberg et al. [16] as “smooth” when the roughness is 0.0–0.4 μm, “minimally rough” when the range is between 0.5 and 1.0 μm, “moderately rough” with a range between 1.0 and 2.0 μm and “rough” when the value is greater than 2.0 μm. Comparing different type of surfaces, it has been demonstrated that a rough surface topography allows a closer contact between blood clots and implant’s surface and increases the bone-to implant-contact. These features allow the migration and differentiation of precursor osteogenic cells, which improves bone apposition [17]. 

More recently, attention has been focused on the production of nano-roughened surfaces, obtained through a variety of processes: the application of a layer of TiO_2_ nanotubes, the coating with hydroxyapatite, calcium-phosphorus compounds or functional peptides, the photofunctionalization with UV rays, the ablation laser or the fluoride treatment by cathodic reduction [18,19,20,21,22,23,24]. The nanostructures increase the surface available for the adhesion of proteins carried by the blood and interstitial fluids and allow a better adhesion of the integrins. Some studies that have evaluated osteoconduction on titanium and have shown better results in terms of growth of osteoblasts in nano-rough surfaces than in micro-rough ones [25].

Surface nanotopography can be an important factor in adding hydrophilicity to implants but it is also influenced by the purity of the surface and the physical-chemical properties [26]. On the surface, pure titanium has a thin (1.5–10 nm) layer of titanium dioxide which is responsible for the migration of calcium and phosphorus ions from the bone matrix onto its own surface [27]. However, the phenomenon of biological aging of titanium leads to the formation of hydrocarbons on the surface due to carbon contamination from the atmosphere. This leads not only to a decrease in hydrophilicity over time but also to a decrease in the ability to attract integrins and osteoprogenitor cells [28].

To obtain a hydrophilic surface performing over time, various techniques have been suggested such as photo-functionalization, treatment with argon plasma and bioactivation of SLA surfaces (sSandblasted, large grit, acid-etched) by drying under nitrogen protection to prevent exposure to air and then storing implants in a sealed glass tube containing isotonic NaCl solution (SLActive) [29]. 

In addition, a dry storage of hydrophilicity of sand-blasted and acid etched surfaces is possible by drying a salt on the surface [30]. The process was studied by Lüers et al. [31] incubating the implants in saline solutions (of which the best in performance was potassium phosphate) for several hours and then air-drying the surfaces for two hours. Therefore, Lüers et al. [32] clearly demonstrated that the preservation of the desiccant layer of the contact angles is based on chemistry and is not due to the saline layer which physically protects from presumed atmospheric contamination.

In this study is evaluated a surface called “ABT” (Alpha-Bio Tec Ltd., Petach Tikva, Israel) obtained from a sandblasting process for the creation of macropores (20–40 microns) and double thermal etching for the creation of micropores (1–5 microns) with the addition of potassium chloride and potassium phosphate salts in order to obtain a nano-roughened surfaces with high wettability and a dry technology. Finally, the same surface was activated by dissolving the salts in distilled water with the aim of obtaining a surface with the same physical and chemical properties as the Nano surface but with a higher surface titanium purity due to the dissolution of salts (Nano H_2_O). The aim of the present study was to microscopically describe the characteristics of this new bioactivated surfaces using a dry technology.

## 2. Materials and Methods

The present in vitro study was designed to analyze the microscopic aspect of three different implant surfaces:

(1) ABT: titanium grade 5 alloy (Ti-6Al-4V) surface obtained from a sandblasting process for the creation of macropores (20–40 microns) and double thermal etching for the creation of micropores (1–5 microns) (Alpha-Bio Tec Ltd.).
(2) Nano: surface obtained by adding salts (potassium chloride and potassium phosphate) to the ABT surface through immersion in a saline solution and subsequently removed from the solution and dried in the air.
(3) Nano H_2_O: a surface activated by the hydration of salts of Nano surface with distilled water.

All surfaces were analyzed as freshly prepared.

### 2.1. Sample Size

A power analysis was estimated on the pilot samples [32] using the mean contamination values of 117.5000 ± 0.0054 spots/field (control) vs. 1,1348.5 ± 0.0007 spot/field (test) (*p* = 0.0001) was projected by setting effect size dz = 1.438, error probability a = 0.05, and power = 0.95 (1-b error probability), resulting in 4 sample from each sub-group (G* Power 3.1.7 for Mac OS X Yosemite, version 10.10.3).

### 2.2. Topographic Analysis

ABT and Nano discs were examined using a Helios Nanolab 600 (FEI Company, Hillsboro, OR, USA) at the electron microscopy laboratory of the Roma Tre University (LIME, Rome, Italy). Nano samples presenting a saline coating were observed both unprocessed and after salt removal by solubilization in distilled water and subsequent drying, in order to visualize both the titanium surface and the salt coating. Disc surfaces were scanned by detecting secondary electrons with an operating voltage of 5 kV and an applied current ranging from 0.86 pA to 0.34 nA. In order to analyze the topographical features on the meso-, micro- and nanoscale, the samples were inspected at different magnifications, varying from 1000× (horizontal field of view of 298 μm) to 500,000× (horizontal field of view of 597 nm). For each disc, micrographs were acquired in randomly selected regions of the sample surface (excluding areas that were clearly non-commensurable) [33].

### 2.3. EDX

Energy-dispersive X-ray spectroscopy (EDS or EDX or EDAX) according to Sawase et al. [34], surface pollution chemical characterization was investigated by energy-dispersive X-ray spectroscopy. EDX spectra of the discs were acquired at an accelerating voltage of 10 kV using a Gemini 300 field emission SEM system (Carl Zeiss AG, Jena, Germany) equipped with an XFlash 6-60 EDS system (Bruker Nano GmbH, Berlin, Germany) at the LIME laboratory, University of Roma Tre. The high energy electrons beam produced by the electron microscope impinge the sample surface and stimulate the emission of characteristic X-rays from the contaminants. The emitted x-rays detected by EDX allow to obtain chemical profiles of the different elements on the implant surfaces. A non-destructive analysis on a microscopic scale was performed. In fact, the atomic concentrations of Ti, O, C and several other elements were examined and the relative concentrations (in atomic %) of the elements was detected. The chemical analysis of contaminants was performed with a minimum 1000 times magnification.

### 2.4. Contact Angle Characterization

Surface wettability was estimated according to Duske et al. [35] by measuring the contact angle with water. Briefly, a drop of deionized distilled water with a volume of 3 µL was gently poured with a micropipette on the tested surfaces. One minute after its deposition, the resulting sessile drop was photographed using an EC3 ccd camera (Leica Microsystems, Wetzlar, Germany) coupled with a Navitar zoom 6000 macro lenses (Navitar, New York, NY, USA). Contact angle was determined using the image analysis software, ImageJ. For each sample, the contact angle measurement was repeated seven times by placing the drop at different spots of the disk in order to test multiple areas of the samples and to prevent any surface alteration by the liquid. To test the nano discs, covered by saline layer, a separate sample was used for each water drop.

### 2.5. Statistical Analysis

Due to the nonparametric nature of the data collected, differences between groups were analyzed using the Kruskal-Wallis test, while the differences were estimated using Man-Whitney U test, by means of GraphPad Prism 6 software (GraphPad Software, Inc., 2015, La Jolla, CA, USA). All of the statistical comparisons were conducted with a 0.05 level of significance

## 3. Results

### 3.1. SEM Results

**Figure 1:**
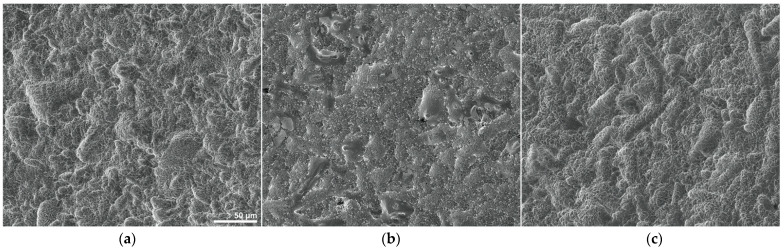
SEM micrographs (1000×) providing an overview of (**a**) ABT, (**b**) Nano and (**c**) Nano H_2_O titanium discs at low magnification (horizontal field width of 298 μm).

At lower magnification (Figure 1) the three different discs (ABT, Nano and Nano H_2_O) show many similarities as their surfaces exhibit a comparable roughness resulting from alternating ridges and valleys, showing similar size and distribution both across the same sample and between different discs.

**Figure 2:**
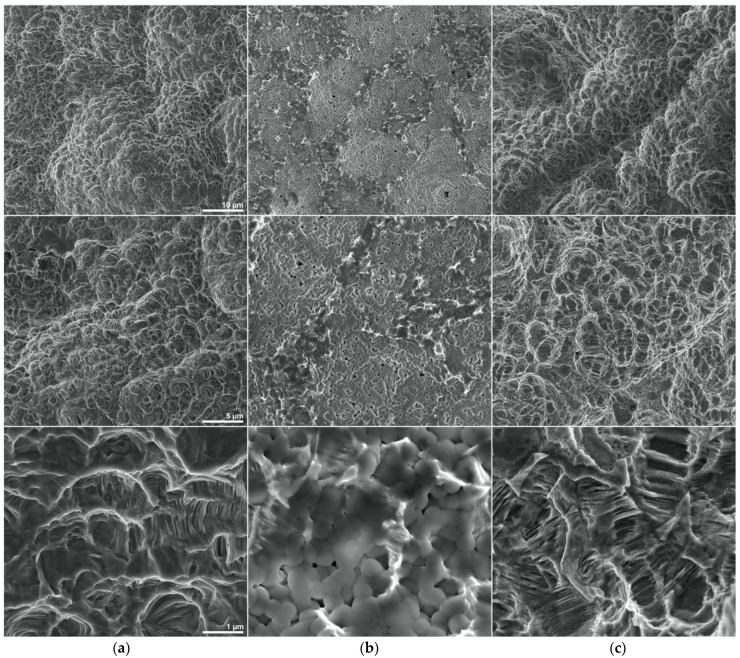
SEM micrographs illustrating the surface topology and roughness of (**a column**) ABT, (**b column**) Nano and (**c column**) Nano H_2_O discs at high magnification (horizontal field widths of 59.7 μm, 29.8 μm and 5.97 μm, respectively). The magnifications are 5000×, 10,000× and 50,000×, respectively for the first, second and third line of photos.

At higher magnifications (Figure 2) it is clearly visible that the coating of the Nano sample hides a large part of the metal surface, masking its true characteristics and features, since most of the small depressions and indentations are filled or covered by the salts, which appear as series of spheroidal structures with a diameter between 300 and 500 nm. Removal of the saline layer shows that, even at the micrometric level, the surface topography of the Nano sample is comparable to that of the ABT samples, as both display the same pattern and succession of microstructures comprising ridges, crests, squamous regions, depressions, indentations and small compartments.

**Figure 3:**
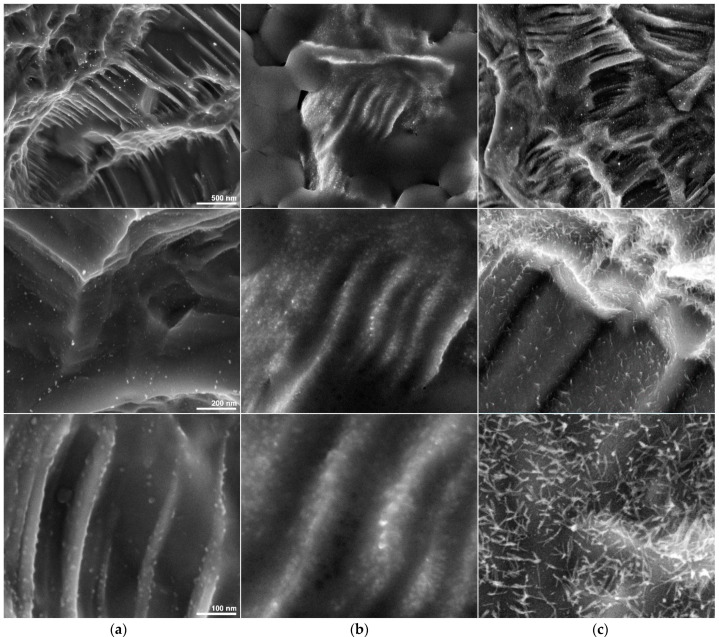
High-magnification SEM micrographs showing the different appearance and density of the nanostructures (horizontal field widths of 2.98 μm 1.19 μm and 597 nm, respectively) of (**a column**) ABT, (**b column**) Nano and (**c column**) Nano H_2_O. The magnifications are 100,000×, 250,000×, 500,000×, respectively for the first, second and third line of photos.

In contrast, when observed at the nanoscale (Figure 3) samples display dissimilar traits, in particular ATB discs present rare and infrequent rounded nanostructures while nanostructured samples feature significantly more nanoparticles with a very different, spiky shape.

The greater density of nanostructures in the nano discs can also be observed before the removal of the salts, in fact in those areas where the titanium emerges and is covered only by a minimal amount of salt, a greater number of nanoparticles can be detected with respect to the ABT sample. 

Interestingly, in this latter case the shape of the surfacing nanostructures appears different from that observed after the removal of the salt (probably due to the persistence of a thin layer of residual coating masking their pointed shape).

### 3.2. EDX Results

**Figure 4:**
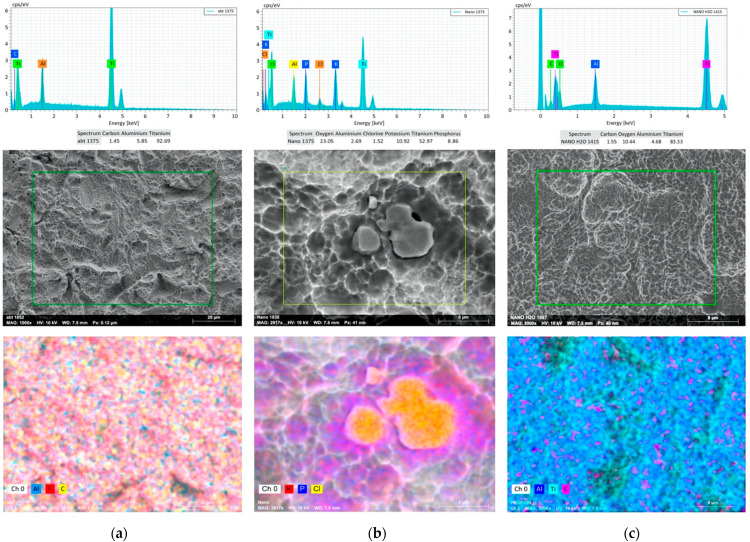
EDX spectra of the different disks samples (ABT (**a**) Column, Nano (**b**) Column and Nano H_2_O after the removal of salts by distilled water, (**c**) Column) and elemental mapping showing the distribution of Al, Ti and C in Abt disks; K, P and Cl in Nano disks and Al, Ti and C in Nano discs after the removal of salts. The magnifications are respectively 1000× for ABT and 3000× for the Nano and Nano H_2_O samples.

EDX elemental analysis (Figure 4) highlighted in ABT disks a predominant presence of titanium and the occurrence in lower percentages of aluminium and carbon, evenly distributed over the sample. Analysis of the Nano discs, in addition to titanium and aluminium, showed the presence of oxygen, potassium, phosphorus and chlorine, the latter being localized in specific regions that are clearly distinguishable in SEM micrographs by element mapping (Figure 2). EDX analysis performed on Nano disks, after removing the saline layer (Nano H_2_O), detected the presence of titanium, oxygen, aluminium and carbon, uniformly spread throughout the sample.

### 3.3. Contact Angle Analysis

**Figure 5:**
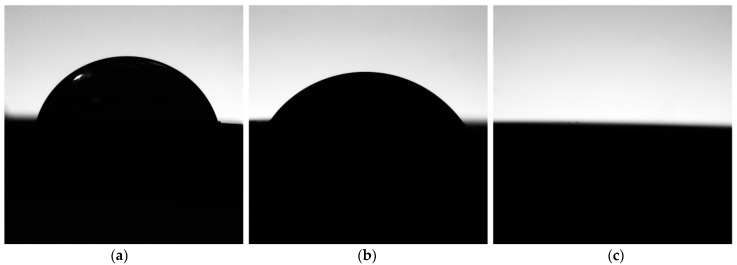
Wettability of different disc samples: ABT (**a**), Nano H_2_O (**b**) and Nano (**c**). The contact angles are respectively: 76.5; 55.3 and 0 (or not measurable).

The contact angle (CA) measurements on all analyzed samples ranged between 76.5° and 0°, which is the minimum angle measurable by the instrument both immediately after treatment and 1 min thereafter. The samples showed values of contact angle of 76.5°, 55.3° and 0° or no measurable, respectively for ABT, Nano H_2_O and Nano (Figure 5). Values remained stable after 1 min. The statistical analysis, carried out considering a value of 10° for the contact angle of all Nano samples, showed a significant difference (*p* = 0.01) between the contact angles measured on ABT, Nano and Nano H_2_O.

## 4. Discussion

The osseointegration process is influenced by the roughness and chemical composition of the implant surfaces which play a central role in the early biological response of the peri-implant tissues [36,37,38]. A specific degree of roughness is essential in the early stages of osseointegration in order to obtain a good bone-implant contact. [39]. The μm roughness should provide better biomechanical interlocking. The clinical importance of nanostructures, on the other hand, is still full of contradictions in the literature [14]. It seems clear that nano-roughness provides more adhesion sites for the initial integrins and filopods that will come into contact with the implant surface [40,41]. Experiments conducted in vitro and in vivo have revealed that nanostructures lead to greater adhesion and proliferation of osteogenic cells [42]. Importantly, the clinical significance of these nanostructures is still unknown [15]. 

In this study it was evaluated what kind of effect salts exert on surface wettability and surface nanotopography. The results showed that it is possible to remove the salts by hydration with distilled water while maintaining a pure surface and with a nano-roughness of the surface. According to the hypothesis of the study, the Nano surface thus hydrated could have a surface similar to ABT in terms of micro-roughness but with different physical-chemical properties.

Salts with diameters between 300 and 500 nm, however, hide the true surface characteristics at the nanometer level, revealing surfaces that are different from ABT. However, if we remove the salts at “Nano”, the surface topography of the Nano sample is comparable to that of ABT samples at a micrometric level. At the nanometer level, ABT presents on the surface rare and infrequent rounded nanostructures while the Nano H_2_O and Nano surfaces demonstrate a greater number of nanoparticles with a very different pointed shape (Figure 1). This greater density of nanostructures can be observed even before the removal of salts, therefore the nano surfaces, regardless of the presence of salts, have a more articulated topography than ABT. In the “Nano H_2_O” surface we have a different surface from the one with the salt because probably the persistence of a thin layer of residual coating masks the pointed shapes of the surface. These nanostructures determine a nanostructured topography (1–100 nm) that not only could increase its surface energy but could provide a structure similar to extracellular matrices that allow protein adhesion by improving the signaling pathways that control cell adhesion, proliferation and differentiation in osteoblasts.

When an implant is inserted into the osteotomy, the first biological event that occurs is the absorption of water molecules and subsequently of proteins from the bloodstream or serum that bind to the hydrated surfaces [43]. In general, as there is no direct binding of the cells to the implant, these proteins, called integrins, play a key role in binding to the implant surface and subsequent cell adhesion, migration, proliferation and differentiation [44]. In the literature it has been shown that a hydrophilic implant surface (SLActive) leads to an increased BIC of 60% two weeks after surgery and speeds up bone maturation, consequently increasing the potential of immediate loading protocols compared to SLA surfaces [45].

The results in term of wettability revealed significant differences between the examined surfaces of the three samples (*p* = 0.01). While the ABT surface shows a CA of 76.5° for which it can be classified as a hydrophilic surface, the modification with salts has made the Nano surface superhydrophilic having a CA of 0°. The removal of the salts has instead made the hydrated surface Nano H_2_O hydrophilic as its CA is 55.3°.

Conserving an activated surface state by processing the implant screws after acid-etching under protective gas and then storing them in saline, argon plasma, alkaline treatments and photolysis upon UV-C are different methods present in the literature to obtain a greater hydrophilicity and a higher surface energy [46,47,48,49,50,51]. 

In this study this new method to obtain a high surface energy and wettability through the use of potassium phosphate and potassium chloride salts was validated. Furthermore, the dissolution of the same in distilled water allowed the evaluation of the activated surface itself by removing the surface salts and therefore increasing the purity of the titanium essential for a correct osseointegration. However, further studies will be needed to understand how long the wettability of the Nano surface will be stable over time.

In this study surface cleanliness was analyzed using SEM and EDX with the aim of obtaining the spectrum of the compounds present on the surface and mapping the distribution of the elements. 

The EDX analysis in particular confirms the results already discussed in literature in a study by Schupbach et al. [52] regarding the ABT surface where a low number of Al atoms were found, suggesting that they were Al_2_O_3_ particle remnants of the blasting process. The analysis of the Nano discs, in addition to titanium and aluminum, highlighted the presence of oxygen, potassium, phosphorus and chlorine, the latter being located in specific regions clearly distinguishable in the SEM micrographs by mapping the elements (Figure 2). These elements in addition to the ABT surface are due to the presence of potassium chloride and potassium phosphate salts. The EDX analysis performed on Nano discs, after removing the saline layer by hydrating the surface with distilled water, revealed the presence of titanium, oxygen, aluminum and carbon, uniformly distributed throughout the sample (Figure 4).

This is a key result of the study as it demonstrates that a surface on which dried salts have been applied to increase its wettability and stability over time, once in contact with an aqueous solution, dissolve completely, exposing the same elements of the ABT surface on the surface.

The characterization of this new Nano surface is very interesting thanks to its nanostructure, its physical-chemical composition and the CA of 0° in the phase with the presence of salts.

However, one of the limitations in understanding how these surfaces work was addressed by a review by Wennerberg et al. in 2019 [14] which revealed that surface topography does not clearly affect the clinical outcome of titanium implants for this reason further studies will be necessary to highlight, where present, significant clinical differences between different surfaces.

## 5. Conclusions

Surface’s topography in nanoscale features of dental implants have a crucial role in cell-surface interaction promoting cell adhesion, proliferation and differentiation. A high energy surface and a hydrophilic implant is important for the absorption of water molecules from bloodstream and subsequently for the adhesion of the cell to the implant binding protein. This study shows the characteristics of a new bioactivated surfaces using a dry technology from a micrometric and nanometric point of view, paying attention on surface wettability with promising results. In order to better understand the influence of the surfaces on the biochemical process of the host further in vivo studies are necessary.

## Data Availability

Data is contained within the article.
